# Detroit Dental Club Post-Graduate Clinics

**Published:** 1918-07

**Authors:** W. Cecil Trotter

**Affiliations:** Toronto


### DETROIT DENTAL CLUB POST-GRADUATE CLINICS

W. CECIL TROTTER, B.A., D.D.S., TORONTO

The Michigan State Dental convention was held at Detroit from Monday, April 8th, to Friday, April 12th. The convention was one of the best conducted meetings the writer has ever attended. Every subject was presented in a most practical way, being illustrated by charts, lantern slides, moving pictures, or clinical demonstrations. Two days of the session were given over to clinics by the Detroit Clinic Club.

### MY IMPRESSIONS OF THE MEETING

1. Comparative promptness of all sessions as compared with most dental meetings.

2. The growing demand for a more exact, comprehensive and scientific diagnosis before going on with our cases.

3. The commencement of a saner view of focal infection and the demand for more definite proofs that all apical areas of rarification, without exception, are really a source of danger.

4. A demand for absolute, definite aseptic precautions in all root canal operations. This involves sterilization by heat of all instruments, cotton, cotton points, paper points, etc., used in canal work.

5. The endorsement of Dr. Howe's suggestion as to the efficacy of silver nitrate in treatment of infected dentine in putrescent roots.

6. The growing faith in the efficacy of prophylaxis in preventing decay and future root trouble.

7. The absolute and definite systems available for the making of perfectly fitting porcelain crowns for all teeth and for the making of either gold or porcelain inlays, all by the indirect method. These systems seem quite within the capabilities of the average assistant.

8. The large number of cases in which jacket crowns are applicable and desirable.

Dr. Elmer S. Best, of Minneapolis, outlined his methods of securing a comprehensive and accurate diagnosis of a case before proceeding with any operation. He advocated radiographing the whole mouth in every case and always doing it by a regular system of blocking. The mouth is blocked out into ten areas as follows:

(1) Upper right three molars.
(2) Upper right bicuspids and cuspid.
(3) Upper incisors.
(4) Upper left bicuspids and cuspid.
(5) Upper left molars.
(6) Lower right three molars.
(7) Lower right bicuspids and cuspid.
(8) Lower incisors.
(9) Lower left bicuspids and cuspid.
(10) Lower left molars.

The radiographs are arranged in two rows, upper and lower, in the above order, thus presenting a complete set of teeth as viewed from the interior of the mouth. The vitality of every tooth at all in doubt, is to be definitely ascertained by thermal or electrical tests, the latter being preferred by Dr. Best. Roots presenting very unfavorable apical areas are to be extracted. Roots presenting less unfavorable apical areas are to be amputated. Roots presenting favorable apical areas are to be thoroughly and aseptically filled.

On the diagnostic charts vital teeth are designated by the X-sign and non-vital teeth by the — sign.

On the charts, the relative solidity, firmness and integrity of each tooth is designated, also the degree of gingivitis present, if any. The relative urgency or otherwise for operative procedures is also designated on the chart. In order to get all this information the use of accurately articulated study models are advocated. Every tooth left or replaced must be conducive to the future health of the patient.

TABLE SHOWING RELATIVE CONDITION OF ANY TOOTH

| | 1 | 2 | 3 | 4 |
|---|---|---|---|---|
| Relative firmness in alveolus........... | | | X | |
| Condition or relative gingivitis......... | | | | X |
| Relative decay ...................... | | X | | |
| Relative condition of fillings.......... | | | X | |
| Condition of pulp or apical areas....... | | | | X |
| Case number .................. | | | | |

If, under these various headings, a tooth was classified as 3 or 4, under several conditions, it would indicate advisability of extraction. If classified under most conditions as 1 or 2, corrective measures would be indicated. The following operative procedures were advocated in root canal treatment:

1. Swabbing the tooth and surrounding tissues with iodine solution.
2. Apply rubber dam which has previously been sterilized by boiling or by immersion in strong disinfectant.
3. Use rubber dam clamp which has been sterilized.
4. Wash tooth with green soap solution or alcohol.
5. Wash cavity with iodine solution.
6. Dissolve out any gutta percha in canals with zylol solution.
7. Use sterilized Rhein picks or probes to open into canals.
8. Enlarge canals with sulphuric acid or phenol sulphonic and the use of files and barbed broaches, especially the short barbed broach. The apical portion of canal is cleaned by means of an "apex curette."

9. Measure with a sterilized diagnostic wire and radiograph, when sure that apex has been reached, apply paper point dressing of Dakins solution or normal salt solution and seal with zinc chloride cement (if not completing operating at first sitting).

10. Remove dressing and ionize.

11. Pump chloroform and chloropercha into canal, resin. Use sterile smooth broaches to do this.

12. Introduce sterilized guttapercha points into chloroform, resin solution in canals.

13. Seal pulp chamber with zinc chloride cement. All cotton pellats, Johnson & Johnson points, Mayo sponges, etc., must be sterilized with dry heat.

A reservation of not less than two hours is recommended as a time allowance for the average root canal operation.

Dr. Custer suggested the use of a mild electric current (from 2 or 3 dry cells) to inform us when we reach the apical foramen. When one electrode is introduced into the canal moistened with alcohol, and the other is held in the hand of the patient, the needle on the millamperemetre is not affected but on reaching the apical tissues beyond the foramen it is immediately deflected.

The average length of a central root is 21 millameters a lateral 22, a cuspid 24 and a bicuspid 20 millameters. By having short broaches, these lengths indicated by 1, 2, 3 or 4 grooves in the handles and using these in the canals for radiographing the apical foramen may be readily located.

### PORCELAIN AND GOLD INLAYS BY INDIRECT METHOD

Impression of cavity is taken by Kerr compound in form of cones. Sheet tin 3-1,000 inch thick is used as a matrix. In bicuspids and molar approximal cavities, a small strip of tin is placed between the teeth and bent backwards over the occlusal surface of the tooth adjacent to the cavity then the two ends of the tin band between the teeth are bent around the outer margins of the cavity to form a box into which the softened impression compound cone is forced. Be sure to pack wet cotton in between the cervical edge of the matrix and the adjacent tooth to prevent the tin being forced beneath the underlying wall of the adjacent tooth. The impression is cooled and removed either alone or with the matrix and trimmed down to regular lines so as to have a more or less regular base. Thin wax is wrapped around it and it is sunk into soft plaster in a wax or rubber ring. An amalgam model is now made in the same way as for porcelain crowns. Then gold may be burnished into amalgam model or be swaged into it, to get gold matrix. The following steps are used in burnishing a platinum matrix:

1. Wet cotton is forced down against floor of matrix with match.
2. Cotton tape stretched over matrix and cotton.
3. Burnish matrix with bare instruments (Reeves Burnishers).
4. Turn or roll over edges of matrix to form collar for stiffening.
5. Annealing and burnishing with rubber tape (surgical tape or inner tube of tire may be used). Wet surface of rubber and when stretched over matrix burnish to place.
6. Paint edges of platinum matrix with shellac to prevent adhering of edges to porcelain when baking.

### PREVENTIVE DENTISTRY

Demonstration of importance of normal occlusion and normal contacts in the healthy condition of the gums and peridental membrane.

Suggested use of five per cent. normal salt solution as a mouth wash also use of sodium bicarbonate solution in warm water to relieve sensitiveness of cervical margins.

One drachm of zinc phenol sulphate dissolved in

16 ounces of U. S. P. antiseptic solution also makes good mouth wash.

Tricalcium phosphate probably least abrasive base for tooth powder.

By dissolving lead, in Faber ink pencil, in water a more permanent disclosing solution can be made than Skinner's iodine solution which soon washes off.

Alphazone prepared by Parke, Davis Co., was recommended for bleaching and removing stains.

Very fine sifted flour of pumice; 3X Silex and oxide of tin moistened with phenol sodique applied with orange-wood sticks was advised for polishing teeth.

Alpine wood sticks recommended to patients along with ribbon floss.

PORCELAIN CROWNS

Prepare molar root end (or other tooth) with cutting burs and stones, dressing down in line with gum and cutting pulp chamber so that the walls are parallel or slightly divergent, also open canals with tapering instruments so as to allow inlay wax to be forced into them to a depth of about one-sixteenth or a quarter of an inch, and yet be readily withdrawn. Cast a slightly pyramidal shaped core out of pure silver (not coin silver) using two sprue wires and cement to place so as to leave a narrow ledge of tooth same width all way around.

JACKET PORCELAIN CROWNS

Where natural crown is badly broken down it was recommended to cast a pure silver core in shape of cone, and cement it to place in pulp chamber.

Select a copper band, slightly larger than the root circumference, and take an impression with Kerr's stick compound, not softening the core of it, and shoving steadily to place.

Wrap copper band impression with this wax (Kerr's), and sink into ring of plaster, using either wax or rubber ring. Immerse in warm water and remove compound and copper band, then pack with some cheap amalgam. First use amalgam containing excess of mercury, afterwards packing in dry amalgam and removing excess of mercury. Cut slot in buccal side of base of amalgam model (for accurate seating in articulator), make a tin (gauge 40 or 3-1000 inch crescent tin, Consolidated Co.) matrix and a 1-1000 platinum matrix, using orangewood stick for burnishing. Cover tin matrix on model roughly with cement, trimming to shoulder. Transfer to mouth and take compound impression with cement seating jacket in place. Now set amalgam model into the compound impression of cement seating model. Insert ordinary pins into cusps of adjacent teeth in compound impression and fill the cusps with cement, then pour with plaster.

Take impression of opposing teeth in mouth with compound, chill, oil surface, and pour with Spence's metal. Roughly burnish tinfoil over biting surfaces of upper and lower models, transfer to mouth, get patient to close into opposing tin, and carefully stick them together, while in correct occlusion, then insert cement and Spence metal models into them and transfer to a Kerr crown articulating form. This method produces a perfectly accurate articulation with indestructible occluding surfaces.

Build base plate wax around collar of platinum matrix below shoulder and surround with paper telescope and pack in basic porcelain body mixed very thin, and dry with Japanese bibulous paper, now build in lighter shade, and trim to a cone shape. This is now ready for the first baking.

### INLAYS

A very satisfactory and accurate method of mounting amalgam model and articulator on a detachable articulator, designed by Dr. Spalding, and manufactured by Kerr, was demonstrated.

By using this articulator frame the model is easily removable to work upon. Contact points are accurately obtainable as well as correct articulation. Several cases can be prepared and articulated on the same frame, as all the parts are interchangeable.

The indirect method seems particularly applicable to any cavity which involves a contour or where a matrix is necessary in getting the impression. For occlusal cavities, and in fact for any four-walled cavity it is difficult to see why the direct method is not much more desirable. —*Oral Health.*

